# Vaccination Status of Horses in Poland Based on an Internet Survey of the Horse Owners

**DOI:** 10.3390/ani15060834

**Published:** 2025-03-14

**Authors:** Marta Rykala, Marcin Jasiak, Artur Niedzwiedz

**Affiliations:** Department of Internal Diseases with Clinic for Horses, Dogs, and Cats, Wroclaw University of Environmental and Life Sciences, pl. Grunwaldzki 47, 50-366 Wroclaw, Poland; marta.rykala@upwr.edu.pl (M.R.); marcin.jasiak@upwr.edu.pl (M.J.)

**Keywords:** vaccination status, prophylaxis, infectious diseases, internet survey, questionnaire, passports, horses

## Abstract

Preventing the spread of infectious diseases in horses requires breaking the chain of infection through appropriate prophylaxis. In Poland, where the horse population reached 273,006 in 2023, vaccinations are voluntary and primarily the responsibility of horse owners, with mandatory influenza vaccinations limited to sport horses. This study aimed to evaluate the vaccination status of Polish horses against infectious diseases through a survey of 980 horse owners and an analysis of 123 horse passports from slaughterhouses. Survey participants represented all provinces, predominantly owning warmblood horses (86%), but also 10% coldblood and 4% ponies or unidentified. Nearly half engaged in equestrian competitions. While over 90% of sport and pleasure horses were vaccinated against key diseases such as equine influenza and tetanus, only 2.4% of slaughter horses had similar prophylaxis. Cold-blooded horses were underrepresented in the survey (10.4%) but made up 68.3% of the analyzed slaughterhouse sample. The findings highlight a significant disparity in vaccination practices, primarily influenced by the horse’s intended use, with sport and leisure horses receiving better preventive care than slaughter horses. These results underscore the need for targeted educational efforts and policy interventions to improve the epizootiological status of Poland’s horse population and ensure a more uniform approach to disease prevention. A limitation of this study is the accuracy of vaccination records in horse passports, as some owners may not have them during vaccination, leading to discrepancies.

## Simple Summary

Infectious diseases in horses can be prevented by breaking the chain of infection, and vaccination plays a key role in this process. In Poland, where there are over 273,000 horses, vaccination is voluntary, except for sport horses, which must be vaccinated against influenza. This study assessed the vaccination status of horses in Poland through a survey of 980 horse owners and an analysis of horse passports at a slaughterhouse. Most survey respondents owned warm-blooded horses, with many participating in equestrian competitions. The survey revealed high vaccination rates among sport and leisure horses but much lower rates among slaughter horses, where only 2.4% were vaccinated against common diseases like influenza and tetanus. In contrast, over 90% of sport horses were appropriately vaccinated. This study highlights a disparity in disease prevention, which, in the case of sport horses, is primarily driven by mandatory vaccination requirements set by sport governing bodies. These findings emphasize the need for better education and outreach to encourage vaccination across all horse populations, reducing the risk of disease outbreaks and improving overall equine health in Poland.

## 1. Introduction

According to the Polish Horse Breeders Association (2023), the horse population in Poland totaled 273,006 in 2023, comprising 49.63% (135,481) cold-blooded horses, 34.2% (93,683) warmblood horses, and 16.17% (43,842) ponies. For comparison, in the 1950s and 1960s, the number of horses in Poland was approximately 2,800,000, largely due to their use in agriculture and national defense. By the 1990s, this number had declined to 941,000, and by 2000, it had dropped further to 550,000 [1].

A general decrease in the population of cold-blooded horses, primarily bred for consumption and further breeding, has been observed—from 223,349 in 2009 to 135,481 in 2018. Meanwhile, the numbers of warmblood horses and ponies in Poland have remained relatively stable during this period [2].

In Poland, vaccines for horses are primarily available for protection against tetanus and influenza. Equine herpesvirus vaccination is also available, along with rabies. Additionally, vaccines against dermatomycosis (ringworm) are accessible, which has both therapeutic and prophylactic properties. Only veterinarians are authorized to purchase and administer these vaccines. Vaccination is voluntary and depends on the owner’s decision. The Polish Equestrian Association mandates vaccination against equine influenza only for sport horses, following a protocol aligned with FEI regulations. This includes two primary doses administered 21–92 days apart, followed by a booster within seven months of the second dose. Subsequent boosters are required every six months. However, for horses competing at the club level in Poland, different vaccination requirements may apply, as in many countries where an annual booster is sufficient for non-elite sport horses. Additionally, there are variations in vaccine protocols depending on the specific licensed vaccines used, even within the framework of FEI regulations [3].

Currently, there are no regulations requiring the vaccination of horses in Poland for infectious diseases other than equine influenza. Moreover, the vast majority of horses in Poland are not used for competitions. Leisure horses and those raised for slaughter are therefore not subject to any preventative health regulations. There is also no centralized electronic database to track vaccinated and unvaccinated horses. Vaccinations are recorded manually in the equine passport, a document used for individual identification and verified by stewards at competition venues. However, many horses, particularly those born before 2009 or those not involved in competitions or slaughter, lack proper identification. This often results in missing vaccination records, even if vaccinations have been performed. The introduction of mandatory microchip identification for all horses, as required by European and national regulations, has significantly improved traceability and reduced the number of unidentified horses. Since 2009, all foals must be microchipped and registered in the national database, ensuring better compliance with health, welfare, and biosecurity measures. Expanding enforcement and awareness of these regulations could further address identification gaps in older or non-competitive horses. Additionally, member states of the European Union have reported significant issues with equine identification documents, including instances of fraud such as exchanging passports between animals [4].

To date, no comprehensive assessment of vaccination status has been conducted for horses in Poland. The absence of an electronic vaccination database hampers the ability to generate reliable statistics on vaccinated and unvaccinated horses. Consequently, the proportion of the horse population protected against infectious diseases remains unknown, raising concerns about their role as potential links in infectious disease transmission chains. While vaccination does not confer complete resistance, as most key vaccines do not induce sterile immunity, it plays a crucial role in biosecurity by reducing clinical signs and virus shedding, thereby limiting disease spread. This highlights the importance of maintaining high vaccination coverage within the equine population to mitigate transmission risks. This issue is particularly significant given the increasing popularity of equestrian sports in Poland, the growing number of sport horses participating in international competitions, and the country’s geographical location [5].

## 2. Materials and Methods

### 2.1. Survey Study Concept and Design

Information about vaccination status among Polish horses was obtained through two methods: a survey addressed to horse owners and an analysis of passports of horses at slaughter.

The survey was conducted between September 2023 and September 2024 and was disseminated electronically via thematic groups on the Facebook social network, equestrian internet forums, and email. Participation in the survey was open to all horse owners without selection criteria, allowing owners of horses from various utility groups across Poland to contribute. We ensured that the survey was completed only once for each horse by relying on the honesty of the respondents. While we acknowledge the potential for duplicate entries, we emphasized the importance of accurate reporting in the survey instructions to encourage truthful responses.

All confidential information obtained from horse owners was handled in accordance with GDPR regulations. The data were collected solely for research purposes and were processed anonymously whenever possible. Personal identifiers were removed or pseudonymized to ensure privacy. Access to the data was restricted to authorized personnel only, and appropriate security measures were implemented to prevent unauthorized access, loss, or disclosure.

Horse passports used in the study were collected from all horses slaughtered on October 25th and 28th, 2023, at the horse slaughterhouse in Rawicz, the largest horse slaughterhouse in Poland, located in the Wielkopolskie Voivodeship. The documents analyzed were issued by one of the five horse breeders’ associations operating in Poland. Vaccination status was assessed for horses originating from 11 of Poland’s 16 provinces, with the origin determined based on the residence of the last owner, including institutions purchasing slaughter horses.

### 2.2. Methodology of the Online Survey

The survey consisted of four thematic sections. The first section focused on the horse owners, who were classified by their region of residence (16 voivodeships), age (five age ranges: 18–25 years, 26–30 years, 31–40 years, 41–45 years, and over 45 years), and the number of horses they owned (five categories: 1; 2–5; 6–10; 11–20; >20). The second section pertained to the horses themselves. Respondents could provide multiple answers for questions concerning horses of different ages, usage types, and preventive care practices. Owners reported the ages of their horses (five age ranges: less than 1 year, 1–5 years, 6–10 years, 11–20 years, and over 20 years), their breeds (warm-blooded, cold-blooded, and ponies), and their transport frequency (five options ranging from at least once a month to no transport at all).

The third section focused on the usage of the horses. Respondents categorized their horses as used for sport, recreation, breeding, or unused. Owners who indicated their horses were used for sport specified the competition level (regional, national, or international). The final section addressed prophylactic practices. Participants were asked whether they implemented preventive measures for all or some of their horses (yes or no) and which diseases their horses were vaccinated against. They could choose from 15 vaccines approved for use in horses available globally. Respondents also reported whether basic vaccinations were performed (for all horses, some horses, none, or expressed uncertainty). Additionally, horse owners identified potential challenges related to vaccination by selecting from six predefined options (cost, arranging veterinary visits, restless horse behavior, post-vaccination complications, adherence to vaccination schedules, or no problems) or providing their own answers. A question on veterinary procedures accompanying vaccination was also included, where participants indicated whether vaccinations were documented in passports or other records, occasionally documented, or not documented due to the absence of a passport.

### 2.3. Evaluation of Vaccinations Based on Horse Passports

A total of 123 passports issued by Polish Horse Breeders’ Associations were analyzed. The analysis included the horses’ place of origin (provinces), age (five ranges: less than 1 year, 1–5 years, 6–10 years, 11–20 years, and over 20 years), and breed (cold-blooded, half-cold-blooded, warm-blooded, ponies, or primitive type). The passports were examined to determine whether the horses had ever been vaccinated against infectious diseases and whether appropriate basic vaccinations had been administered.

## 3. Results

### 3.1. Demographic Profile of Horse Owners and Equine Characteristics

A total of 980 individuals from across Poland participated in the study, nearly 500 of whom declared involvement in equestrian competitions and responded to questions regarding sport horses. The largest demographic group, representing 33.9% of respondents, was aged 26–35 years. A slightly smaller group, 32.6%, were aged 18–25 years. Individuals aged 36–45 years constituted 15.2%, those under 18 years accounted for 10.2%, and respondents over 45 years made up 8.2%.

Respondents from the Dolnośląskie Voivodeship formed the largest group, representing 19.5% of participants, followed by Silesian residents at 15.9%. Significant representation was also noted from Wielkopolskie (11.2%) and Mazowieckie (10%). Respondents from other voivodeships contributed percentages below 10%, with the highest being Warmińsko-Mazurskie (8.3%) and the lowest being Podlaskie (0.9%). Four neighboring non-EU countries were also represented in the survey: Warmińsko-Mazurskie, Podlaskie, Lubelskie, and Podkarpackie.

Nearly half (48.2%) of the respondents reported owning a single horse. A further 35.1% owned between two and five horses, while only 8.2% reported having six to ten. Owners of larger herds (11–20 horses) comprised 4.7% of the participants, and those with over 20 horses accounted for 3.9%.

The majority of horses owned by respondents fell within the middle age range, with 40.0% aged 10–20 years, followed by 28.0% aged 5–10 years, 18.0% aged 1–4 years, 10.0% over 20 years old, and 4.0% being foals under one year of age. Warmblood breeds were the most commonly owned, with 86% of respondents reporting ownership. Cold-blooded horses were reported by 10% of participants, while ponies accounted for 4%.

### 3.2. Utilization and Transport History of Horses

**Figure 1:**
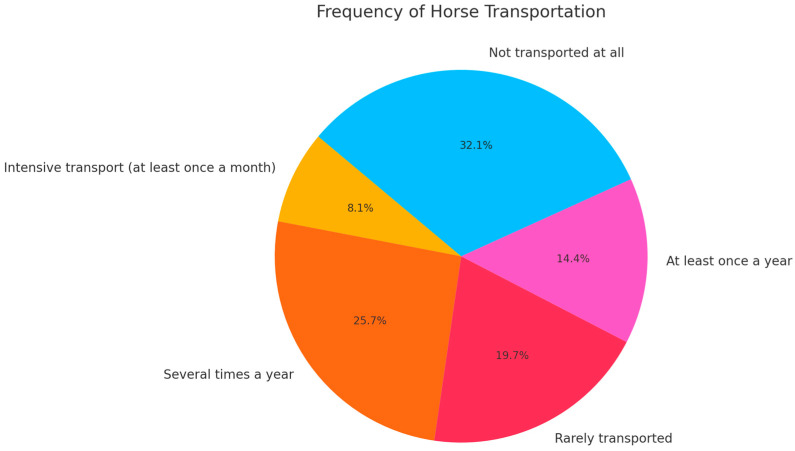
Frequency of horse transportation.

Regarding horse transportation, 8.1% of the surveyed horses were transported at least once a month, while 25.7% were moved several times a year. Horses that were rarely transported accounted for 19.7%, and those transported at least once a year constituted 14.4%. A significant portion (32.1%) were not transported at all (Figure 1).

Recreational use of horses was reported by 60.2% of respondents, while 50.7% stated their horses were used for sport. Breeding purposes were reported by 21.2%, and 10.2% of respondents indicated that their horses were not used in any specific way. The combination of sport and recreation horses exceeding 100% is due to the fact that many horses are used for multiple purposes and respondents were allowed to select multiple answers.

Out of the 980 respondents, 497 stated that their horses were used in sport. Among them, the majority (86.2%) participated in regional-level competitions, 31% in national-level competitions, and 10% in international events. Similarly, the total percentage for horses competing at different levels exceeds 100% because some horses compete at more than one level and respondents were allowed to select multiple answers.

### 3.3. Declared Equine Vaccination Practices Based on Survey Data

**Figure 2:**
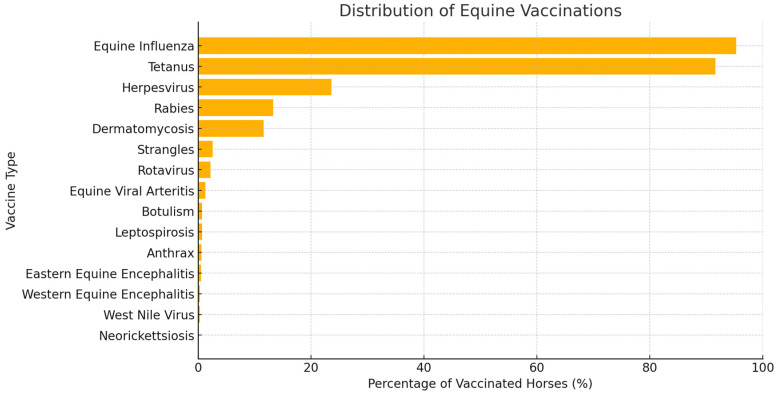
Distribution of equine vaccinations.

A majority of respondents (83%) reported implementing preventative measures against infectious diseases for their horses. However, 11.5% did not perform any preventative measures, and 5.5% applied such measures selectively. Vaccinations against equine influenza (95.3%) and tetanus (91.6%) were the most commonly reported, followed by herpesvirus (23.6%). Vaccinations against rabies (13.3%) and dermatomycosis (11.6%) were also relatively common. Other reported vaccinations included strangles (2.6%), rotavirus (2.2%), equine viral arteritis (1.3%), botulism (0.7%), leptospirosis (0.7%), anthrax (0.6%), Eastern equine encephalitis (0.5%), Western equine encephalitis (0.3%), West Nile virus (0.3%), and neorickettsiosis (0.1%) (Figure 2).

Of those who implemented specific preventative measures, 75.2% indicated that they began with basic vaccinations for all their horses, while 7.3% applied them to some animals only. A notable proportion of respondents (10.5%) were unsure about basic vaccinations, and 5% were unfamiliar with the term. Only 2% of respondents indicated that basic vaccinations were not performed for their horses.

Most respondents (86.9%) confirmed that vaccinations were documented in the horse’s passport, 10.6% noted that this was not always carried out, and 0.9% reported documenting vaccinations outside of the passport. A small group admitted that they did not maintain documentation (0.6%) or lacked a passport (0.6%). Additionally, 0.4% of respondents were unaware of whether the veterinarian recorded vaccinations in the passport.

**Figure 3:**
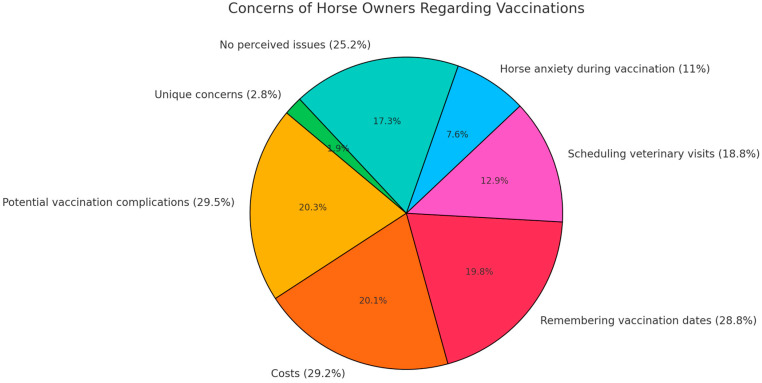
Concerns of horse owners regarding vaccinations.

Despite growing awareness of the importance of preventing infectious diseases and the availability of vaccines, some respondents highlighted challenges in using them. Concerns included potential vaccination complications (29.5%), costs (29.2%), and the need to remember upcoming vaccination dates (28.8%). Other challenges included scheduling veterinary visits (18.8%) and horse anxiety during vaccination (11%). However, 25.2% of respondents did not perceive any issues with specific preventative measures, while 2.8% cited unique concerns, such as the frequency of vaccinations for sport horses, mandatory vaccination regulations by the Polish Equestrian Association, limited availability of monovalent vaccines, and the requirement to use polyvalent vaccines. Some also expressed concerns about potential adverse effects, such as weakened immunity, harmful vaccine components, and virus mutations (Figure 3).

Among the 123 horse passports reviewed for slaughtered animals, cold-blooded horses constituted 68.3%. Warmblood breeds accounted for 13%, ponies and small horses for 5.7%, and primitive breeds for the remainder. Most slaughtered horses were young, with 16.3% under one year old and 35.8% between 1 and 4 years. Horses aged 5–10 years made up 7.3%, those aged 10–20 years comprised 30%, and 3.3% were over 20 years old. The date of birth was unknown for 7.3% of horses. Most slaughtered animals originated from Wielkopolskie (37.4%) and Pomorskie (20.3%), followed by Kujawsko-Pomorskie (12%), Mazowieckie (11.4%), and Lubelskie (11.4%). Fewer animals came from Dolnośląskie (2.4%), Podkarpackie (1.6%), and Małopolskie, Podlaskie and Śląskie (0.8% each).

Of the slaughtered horses, only three (2.4%) had documented vaccinations for influenza and tetanus, including basic vaccinations. These horses were thoroughbreds registered with the Polish Jockey Club, suggesting they were likely used in sport and transported frequently. One horse (0.8%) had documented vaccination against EHV. Additionally, 2.4% of horses had vaccinations for influenza and tetanus without basic vaccinations. A further 6.5% received a single polyvalent vaccine dose for influenza and tetanus, while 0.8% were vaccinated against influenza only. In total, 13% of slaughtered horses had ever undergone specific preventative measures.

## 4. Discussion

The survey results raise a question of whether the study reached a representative group of recipients. Younger individuals, who are more likely to use social networking sites and online communication, were the most frequent respondents. Despite the widespread availability of the internet, the survey might have been less accessible in rural areas. The data also correlate with the authors’ workplaces and efforts to promote the study among local horse owners in the Dolnośląskie and Śląskie Voivodeships. Moreover, for the Mazowieckie and Wielkopolskie Voivodeships—ranked first and third in terms of the highest number of Equidae in Poland—the results are consistent with regional patterns. This geographic distribution was reflected in the findings, with a relatively small proportion of respondents coming from the eastern voivodeships, which are less urbanized compared to the rest of the country. The general profile of horses evaluated in the study revealed a predominance of warmblood horses, typically young or middle-aged, actively used for recreation or regional-level sports. Horses used in these activities are at risk of sanctions if vaccination requirements at competitions are not met. Notably, some respondents stated that they vaccinate their horses solely due to regulatory requirements, reinforcing the notion that usage influences preventive measures and that enforcement is a key motivator for compliance. Conversely, many horses included in the survey were breeding animals or older individuals over 20 years of age, often retired or rarely moved, thus with lower perceived vaccination needs. Research from the United Kingdom and Finland aligns with these findings, showing that a horse’s lifestyle significantly influences preventive care. Owners tend to either avoid vaccinating or no longer consider it necessary for horses that do not interact with others, are stationary, or are considered too old. This decision is often based on the perception that these horses have a lower risk of exposure to infectious diseases. However, it is important to emphasize that even isolated or older horses may still benefit from vaccination, particularly for diseases with environmental transmission routes, such as tetanus and influenza. The spread of influenza, as seen in outbreaks like the one in Australia, can have significant health and economic consequences, highlighting the importance of vaccination even in seemingly low-risk horses [6,7].

Regarding the credibility of the results, it is important to consider the age of the surveyed horses in relation to vaccination protocols. Some horses under one year of age were included in the dataset, raising questions about their vaccination status. While foals generally begin their primary vaccination course around six months of age, once maternal antibodies wane, the presence of very young horses in the survey suggests the need for further clarification. A more reliable analysis would involve categorizing horses younger than six months separately to account for those not yet eligible for routine vaccinations. Additionally, it would be valuable to determine whether these younger animals had already received their primary vaccinations, such as for influenza and tetanus, to better assess vaccination coverage within this group. The current statistics from the Polish Association of Horse Breeders indicate a higher population of cold-blooded horses compared to warmbloods and ponies. However, the survey likely did not adequately reach cold-blooded horse breeders. This may be due to the specific use of these animals, as breeders might not perceive studies on infectious disease prevention as relevant. In 2004, Poland produced one-third of the 69,000 t of horse meat in the European Union, primarily from cold-blooded horses [8]. These horses are often kept in one location throughout their lives, with displacement occurring only during their final transportation for slaughter. Slaughterhouses, such as the one in Rawicz, operate with regional purchasing patterns, which may not represent all parts of the country. The analysis of passports obtained from only two slaughter days further limits the generalizability of findings, particularly regarding horses from the Lubelskie, Podlaskie, and Warmińsko-Mazurskie Voivodeships, where extensive breeding is more common. The overwhelming majority of respondents reported adhering to proper infectious disease prevention protocols. However, discrepancies in the responses—such as 813 affirmative answers regarding disease prevention versus 878 declarations of specific vaccinations—suggest that some respondents may not fully understand the definition of prevention. Additionally, several respondents provided vague or incomplete answers, such as “none of the above”, “standard three-in-one”, or “the veterinarian decides”. Some even mentioned vaccines unavailable in Poland, possibly due to direct imports or vaccinations administered outside the European Union. These findings highlight a lack of awareness among some owners about their horses’ vaccination status. On a positive note, many respondents rely on veterinarians to make preventive care decisions, which is especially relevant for less common vaccinations, such as against herpesvirus for traveling horses or rabies for free-range herds.

Responses regarding vaccination challenges indicate increasing awareness among horse owners. For instance, many are aware that vaccinating against tetanus more frequently than every two years is unnecessary. However, a concerning trend is the growing skepticism about vaccine safety, including misconceptions about harmful substances, which mirrors similar issues in human medicine.

Despite the mandatory reporting of adverse events by veterinarians and the publicly available data on vaccine safety provided by the European Medicines Agency, there remains a discrepancy between documented adverse reactions and horse owners’ perception of perceived complications. While the Summary of Product Characteristics for commonly used vaccines indicates that adverse reactions are rare, vaccine hesitancy may persist due to anecdotal experiences, misinformation, or limited awareness of official pharmacovigilance data. To address this issue, improved communication between veterinarians and horse owners is essential, highlighting the reliability of official safety reports and promoting evidence-based decision making. Furthermore, simple tools available in veterinary practice, such as vaccination reminders and owner education initiatives, could enhance compliance and confidence in vaccination programs. Additionally, concerns related to weakened immunity, harmful vaccine components, and virus mutations require clarification. There are no modified live vaccines for the most common equine infectious diseases on the EU market. The only modified live influenza vaccine, available for intranasal use in the U.S., is not widely used, and in the EU, a modified live *Streptococcus equi* vaccine exists but is also not commonly administered. Ensuring clear and accurate information about vaccine composition and safety is crucial in addressing misconceptions and improving vaccination coverage in the equine population.

In the EU, monovalent vaccines are available for key equine infectious diseases, including influenza, tetanus, equine herpesvirus, *Streptococcus equi*, and West Nile virus. Contrary to the perception of limited availability, the only commonly used polyvalent vaccines in Europe are those combining influenza and tetanus. In contrast, larger combination vaccines, which include multiple antigens in a single dose, are available in the U.S. but are not licensed for use in Europe. The requirement to use polyvalent vaccines is, therefore, not a limitation in the EU, as veterinarians and horse owners can tailor vaccination protocols using monovalent options to meet individual health and risk considerations. This distinction highlights the importance of regional regulatory differences in vaccine availability and selection.

Economic factors also play a significant role in vaccination decisions, particularly for low-value horses. The cost of vaccination remains an important factor influencing owners’ decisions. In Poland, the price of an influenza–tetanus polyvalent vaccine ranges from PLN 60 to 100, excluding the cost of a veterinary visit, which varies by region and the number of horses vaccinated. While this cost may be significant for some owners, particularly those raising draft horses where the market value is relatively low (approximately PLN 4540), it is less likely to be a limiting factor for owners of sport horses. These individuals already invest considerable amounts in equipment, farriery, competition fees, and transportation. Additionally, the financial burden of vaccination should be considered in the context of the demographic profile of respondents, as many were young urban horse owners. Future studies could provide more precise insight by assessing the median income of this group and its impact on vaccination decisions.

For breeders raising nine cold-blooded horses, the profit from selling one horse would cover the vaccination costs for the entire herd. However, additional costs, such as deworming and unexpected treatments, may deter owners from investing in proper prophylaxis. The survey primarily captured data from young urban residents who likely recognized the value of their responses. Promisingly, this group demonstrated significant awareness of the importance of preventing infectious diseases in horse health. Nearly half of the respondents owned only one horse, consistent with earlier findings that vaccination rates decrease as the number of owned horses increases. Sport and transport horses had higher vaccination rates, ensuring better population safety against infectious diseases. However, the findings from the slaughter horse passport analysis diverged significantly from survey responses, supporting the hypothesis that an animal’s intended use heavily influences investment in preventive care [9]. The lack of vaccinations in slaughter horses poses a potential threat in the event of an outbreak. While many of these animals are young, they are still exposed to pathogens from birth. Initial protection is provided by maternal antibodies, assuming the mare is vaccinated or has had prior exposure to specific pathogens. However, as maternal immunity wanes, unvaccinated young horses may become susceptible to infectious diseases, particularly in environments where they come into contact with other animals. This highlights the importance of considering vaccination strategies even for horses with a short production lifespan to reduce potential disease transmission risks.

Increasing vaccination rates is a fundamental objective; however, addressing the underlying factors identified in this study is essential for achieving long-term improvements in vaccine coverage. Owner education is a key component, particularly in enhancing awareness of risk-based vaccination strategies, vaccine safety, and the broader benefits of immunization. Additionally, strengthening biosecurity measures remains critical. Tetanus vaccination is essential due to the high fatality rate of the disease, while comprehensive influenza vaccination programs are necessary to protect both individual horses and the equine industry as a whole. Large-scale outbreaks, such as those reported in France prior to the London Olympics, underscore the economic and epidemiological consequences of inadequate vaccination coverage [10]. These events demonstrate how vaccinated horses can introduce the virus into populations with heterogeneous immunity, including unvaccinated foals and young stock, thereby exacerbating disease transmission. Such findings highlight the importance of targeted vaccination strategies to mitigate the risks associated with infectious disease outbreaks in equine populations. Finally, while falsification of documentation remains a concern, the microchip number is the most difficult aspect to falsify, providing a reliable means of identification. Integrating an electronic database for veterinary medicinal products with horse identification documents could further enhance disease prevention efforts and improve equine safety across the European Union. Although some databases, such as Equitrace, already exist, broader implementation and integration with national systems could strengthen traceability. However, rather than overemphasizing falsification, it is crucial to focus on addressing owner perceptions and barriers that may prevent vaccination, as these factors have a more direct impact on vaccination coverage and equine health. Public education efforts should emphasize collective responsibility for animal health, especially as outbreaks of equine infectious diseases continue to occur globally. The greatest risk to European horses comes from endemic diseases that can be effectively prevented through vaccination, as most horses do not travel. While West Nile virus has been present in Europe for over 20 years, the primary focus should remain on diseases like influenza and tetanus, for which effective vaccines are widely available in the EU [11]. Prioritizing vaccination against these endemic threats is essential for maintaining equine health and biosecurity [12].

A potential limitation of this study is the accuracy of vaccination records documented in horse passports. While passports are required to be updated during vaccination, in practice, some owners may not have the passport with them at the time of vaccination, leading to discrepancies between official records and actual vaccination status. This could result in underreporting or incomplete data in passport-based assessments. Future studies should consider verifying vaccination status through veterinary records or direct owner declarations to improve data reliability.

## 5. Conclusions

This study assessed the vaccination status of horses in Poland, revealing significant disparities based on their intended use. While over 90% of sport and leisure horses were vaccinated, only 2.4% of slaughter-bound horses received prophylaxis, highlighting the influence of regulatory requirements and economic considerations.

Vaccine hesitancy, misconceptions about adverse effects, and perceived low risk for stationary or older horses remain challenges. Discrepancies in reported vaccination rates suggest gaps in owner understanding, emphasizing the need for better education and veterinary guidance. Integrating vaccination records with microchip identification could enhance traceability and compliance. Prioritizing immunization against endemic diseases, such as influenza and tetanus, is essential for improving equine health and biosecurity.

## Data Availability

The data presented in this study are available on request from the corresponding author.

## References

[1] Polish Horse Breeders Association The Horse Population in Poland. https://www.pzhk.pl/hodowla/poglowie-koni-polsce/.

[2] Central Equidae Database The Number of Equidae. https://www.pzhk.pl/hodowla/statystyka-cbdk/.

[3] Polish Equestrian Association Development Strategy of Polish Horse Riding in the Years 2019–2022. https://pzj.pl/sites/default/files/%20Strona%20WWW.%20PZJ.Nowelizacja%2010.05.2019%20r.%20MSiT%20STRATEGIA%20ROZWOJU%20POLSKIEGO%20%20%20JE%C5%B9DZIECTWA%202019-2022.pdf.

[4] Official Journal of the European Union Commission Implementing Regulation (EU) 2015/262 of February 17, 2015, Laying Down, Pursuant to Council Directives 90/427/EEC and 2009/156/EC, Rules on Methods for the Identification of Equidae. https://www.pzhk.pl/wp-content/uploads/Rozporzadzenie_UE-2015_262.pdf.

[5] Polish Equestrian Association Veterinary Regulations of the Polish Equestrian Federation. https://pzj.pl/sites/default/files/przepisy/Przepisy%20Weterynaryjne_2.pdf.

[6] Koskinen H.I. (2014). A Survey of Horse Owner’s Compliance with the Finnish Vaccination Program. J. Equine Vet. Sci..

[7] National Equine Health Survey (NEHS) *018 Report. https://www.bluecross.org.uk/national-equine-health-survey-0.

[8] Types of Horse Use in Poland—Meat Use. http://konie-pkz.dzs.pl/rodzaje-uzytkowania/miesne/.

[9] Central Statistical Office Salaries in Poland According to Data from the Central Statistical Office. https://stat.gov.pl/.

[10] Paillot R., Pitel P.-H., Pronost S. (2019). Florida Clade 1 Equine Influenza Virus in France. Vet. Rec..

[11] Koch R.T., Erazo D., Folly A.J., Johnson N., Dellicour S., Grubaugh N.D., Vogels C.B. (2024). Genomic Epidemiology of West Nile Virus in Europe. One Health.

[12] Cowled B., Ward M.P., Hamilton S., Garner G. (2009). The Equine Influenza Epidemic in Australia: Spatial and Temporal Descriptive Analyses of a Large Propagating Epidemic. Prev. Vet. Med..

